# Joint Task Offloading and Resource Allocation for Intelligent Reflecting Surface-Aided Integrated Sensing and Communication Systems Using Deep Reinforcement Learning Algorithm

**DOI:** 10.3390/s23249896

**Published:** 2023-12-18

**Authors:** Liu Yang, Yifei Wei, Xiaojun Wang

**Affiliations:** 1 Beijing Key Laboratory of Work Safety Intelligent Monitoring, School of Electronic Egineering, Beijing University of Posts and Telecommunications, Xitucheng Road No. 10, Beijing 100876, China; oyangliu@bupt.edu.cn (L.Y.); weiyifei@bupt.edu.cn (Y.W.); 2 School of Electronic Engineering, Dublin City University, Collins Avenue Extension, D09 D209 Dublin, Ireland

**Keywords:** integrated sensing and communication, intelligent reflecting surface, deep reinforcement learning, resource allocation

## Abstract

This paper investigates an intelligent reflecting surface (IRS)-aided integrated sensing and communication (ISAC) framework to cope with the problem of spectrum scarcity and poor wireless environment. The main goal of the proposed framework in this work is to optimize the overall performance of the system, including sensing, communication, and computational offloading. We aim to achieve the trade-off between system performance and overhead by optimizing spectrum and computing resource allocation. On the one hand, the joint design of transmit beamforming and phase shift matrices can enhance the radar sensing quality and increase the communication data rate. On the other hand, task offloading and computation resource allocation optimize energy consumption and delay. Due to the coupled and high dimension optimization variables, the optimization problem is non-convex and NP-hard. Meanwhile, given the dynamic wireless channel condition, we formulate the optimization design as a Markov decision process. To tackle this complex optimization problem, we proposed two innovative deep reinforcement learning (DRL)-based schemes. Specifically, a deep deterministic policy gradient (DDPG) method is proposed to address the continuous high-dimensional action space, and the prioritized experience replay is adopted to speed up the convergence process. Then, a twin delayed DDPG algorithm is designed based on this DRL framework. Numerical results confirm the effectiveness of proposed schemes compared with the benchmark methods.

## 1. Introduction

The integrated sensing and communication (ISAC) framework has been proposed as one of the critical technologies in the six-generation (6G) networks, enabling many emerging applications such as virtual reality, smart city, autonomous driving, etc. [1]. The application scenarios mentioned above require a high data transmission rate while ensuring target sensing performance. In recent works [2,3,4,5], a tight combination of sensing and communication functions in ISAC systems has been achieved through a series of designs, including integrated architecture, waveforms designing, etc. By achieving the sharing of spectrum and wireless infrastructure, the ISAC technology improves resource efficiency and utilization, and reduces signal interference and hardware overhead [6].

However, despite the enormous benefits of ISAC technology, its applications face considerable challenges in practice due to the obstacles of dense buildings or landscape trees in urban environments [7]. Unlike communication systems in which both line-of-sight (LoS) and non-LoS (NLoS) links can be leveraged for data transmission, the radar sensing function relies on the LoS link between the transmitter and the target area, while the NLoS link is considered to be an interference [8]. Therefore, exploring the target sensing problem for the ISAC system without an LoS link is necessary [9].

The intelligent reflecting surface (IRS) is a promising technology in next-generation wireless systems due to its excellent ability to reconstruct wireless environments [10]. By manipulating the phase shifts and amplitude of reflecting elements, the IRS creates the virtual LoS link in NLoS areas. Motivated by the advantages of IRS in reconstructing the wireless propagation environment, it is natural to exploit IRS to assist ISAC systems to improve communication data rate and enhance sensing accuracy and resolution [11]. In the IRS-assisted ISAC system, multiple beams can be synthesized for the user and the desired signal can be enhanced by the joint design of phase shift and transmit beamforming [9,12]. Moreover, the IRS reduces hardware and energy overhead using low-cost passive components without needing a radio frequency (RF) unit [13]. Hence, high spectrum efficiency and low cost advantages prompt us to research IRS-assisted ISAC systems.

Although the IRS-assisted ISAC system shows significant potential, its implementation still faces challenges, such as the joint design of phase shift and beamforming matrices. The ISAC system’s data calculation and signal processing are generally complex and require more resources. Due to the constrained computation and energy resources of the user terminal, the heavy sensory data processing load of user equipment (UE) is solved by mobile edge computing (MEC) technology. MEC works by offloading the computational task from UE to the edge network and achieving better time efficiency and performance [14]. This work investigates the joint resource allocation and task offloading optimization problem in the multi-user IRS-assisted ISAC scenario. In particular, power and spectrum resources are allocated by beamforming and phase shift design, while computing resources are allocated by task offloading.

### 1.1. Related Works

Adopting the IRS to improve communication quality has provided certain benefits; inspired by this, researchers have conducted extensive studies to explore the potential of employing IRS in ISAC systems [8,13,15,16,17,18,19,20,21]. In [8], the virtual LoS channel was created with the IRS’s assistance to enhance the communication and sensing performance in an ISAC system, and the semi-definite relaxation (SDR) was adopted for the beampattern gain maximization problem. The authors in [13] exploited the IRS to strengthen the radar detection function in the dual-function radar and communication system, in which a joint optimization of precoding and IRS phase shift matrices was proposed, and a majorization—minimization (MM) method was used to solve it. A hybrid IRS model was investigated in [15], which comprised active and passive elements for enhancing ISAC systems and realizing worst-case target illumination power maximization through an alternating optimization (AO) algorithm. In [16], the authors proposed an IRS-aided radar system architecture and studied the benefits of IRSs and the deployment location issues. Through a joint beamforming design, the authors in [17] optimized the total transmit power while meeting signal-to-interference-plus-noise (SINR) requirements for communication and radar signal cross-correlation pattern constraint for sensing in IRS-assisted ISAC systems. The authors in [18] proposed penalty dual decomposition (PDD) and block coordinated descent (BCD) methods for the joint optimization problem in the IRS-aided communication radar coexistence system. In [19], the authors studied the joint waveform and discrete phase design in the IRS-aided ISAC system to mitigate the multi-user interference. In [20], an alternative direction method of multipliers (ADMMs) and MM approaches were proposed to optimize the sensing performance while satisfying the communication requirements. The authors in [22] developed an ISAC-assisted MEC and employed IRS to reduce the mutual interference between MEC offloading transmission and radar sensing, and a BCD algorithm was employed. Inspired by the above-mentioned work, we investigate the joint computation offloading and resource allocation problems in the IRS-aided ISAC system.

Recently, the excellent performance of artificial intelligence (AI) algorithms in dealing with nonlinear and high computational complexity problems has triggered a revolution in the industry and academia [23,24,25,26]. Considering that there are numerous elements in the IRS-assisted ISAC system, the high-dimensional optimization problems in this system are difficult to solve using traditional mathematical methods. However, it is very suitable for AI technology. Meanwhile, deep reinforcement learning (DRL) takes advantage of deep learning in neural network training and the extraordinary ability of reinforcement learning on large-scale non-convex problems [25]. Therefore, DRL finds a broad array of applications within the domain of wireless communications, including computing offloading [27], power allocation [28], task scheduling [29], etc. The authors in [30] designed a DRL approach to address a joint transmit precoding and phase shift matrix design with the maximizing data rate optimization goal. An adaptive DRL framework twin delayed deep deterministic policy gradient was developed in [31] to deal with the joint beamformer design problem in IRS-aided wireless systems. The authors in [6] designed a distributed reinforcement learning scheme for the joint optimization problem in the terahertz band IRS-aided ISAC system. Therefore, given the time-varying channel conditions and dynamic resources, we reformulated the proposed optimization problem in our work as a Markov decision process (MDP). Then, an innovative DRL-based framework is developed for solving the joint resource optimization and computation offloading problem. Table 1 lists the main closely-related existing efforts and compares them with our work.

### 1.2. Contributions

We investigate the joint optimization problem in the multi-user IRS-assisted ISAC system. Specifically, the design of transmit beamforming and IRS phase shift matrices for communication and radar sensing, as well as the computation offloading for local data processing, are studied in this context. Our aim is to optimize the system’s data transmission and energy efficiency while meeting the radar sensing requirement and power constraints. Considering the dynamic environment and high-dimensional solution space of the optimization problem, we develop a DRL scheme for solving it. We can summarize the contributions as follows:

- We propose the IRS-assisted ISAC framework, exploiting the IRS to assist and enhance sensing and communication functions in NLoS coverage areas. We construct a comprehensive optimization goal, covering the sensing, communication, and computation offloading. The main goal is to maximize the data sum-rate while minimizing energy consumption under the radar performance, transmit power budget, and offloading time delay constraints through the joint design of transmit beamforming and IRS phase shift.
- Considering the coupled relationship between optimization variables, the joint optimization problem is NP-hard and non-convex, making it challenging to use traditional mathematical methods. Therefore, the optimization problem is formulated as an MDP problem, and two innovative DRL schemes are designed to solve it. Due to the continuous and large-dimension action space, we develop a deep deterministic policy gradient (DDPG) scheme, which combines prior experience replay technology to enhance training efficiency. Furthermore, a twin delayed DDPG (TD3) scheme is designed based on the DDPG framework.
- Simulation results confirm the effectiveness and convergence of our proposed scheme. In contrast with benchmarks, our proposed DRL scheme achieves a better balance between communication and sensing performance. Moreover, system’s energy consumption and latency are optimized by proper computation offloading. Finally, the benefits and feasibility of the IRS-assisted ISAC framework are verified.

Notation: Bold uppercase and lowercase letters represent matrices and vectors, respectively. ${\left(\cdot \right)}^{T}$ and ${\left(\cdot \right)}^{H}$ denote the transpose and Hermitian transpose operators. $\mathrm{Tr}\left(\cdot \right)$ is the rank operation. $\mathrm{diag}\left(\cdot \right)$ expresses the diagonal elements. ${∥\cdot ∥}_{F}$ and $\left|\cdot \right|$ are the Frobenius norm and absolute operators.

## 2. System Model

**Figure 1:**
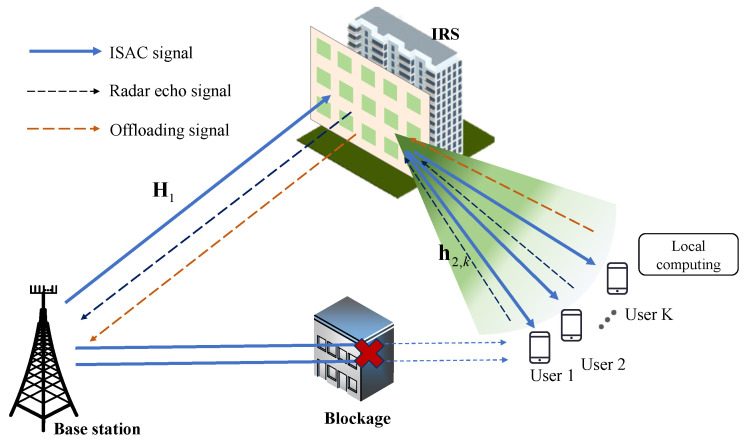
System model.

A multi-user, single-input, single-output (MISO) IRS-aided ISAC system is presented in Figure 1, with *K* single-antenna users and a base station (BS) equipped with *M* antennas. Specifically, the BS deployed the uniform linear array (ULA) antennas, and the IRS employed the uniform planar antenna (UPA). Our work considers a case wherein direct links of BS users are obstructed by dense obstacles. Therefore, the IRS with N×N reflecting elements is employed to aid the user’s wireless data transmission and to provide target sensing service in NLoS areas. We can denote the set of users, BS antennas, and IRS elements as $K=\left\{1,2,\cdots ,K\right\}$, $M=\left\{1,2,\cdots ,M\right\}$, and $N=\left\{1,2,\cdots ,N\right\}$, respectively. The transmitted information-bearing symbol vector is denoted as $s\left(t\right)={\left[s_{1}\left(t\right),\cdots ,s_{K}\left(t\right)\right]}^{T}\in C^{K\times 1}$. The signal transmitted by BS is given by

(1) x(t)=Ws(t),

where $W=\left[w_{1},w_{2},\cdots ,w_{K}\right]\in C^{M\times K}$ represents the transmit beamforming matrix, with $w_{m}\in C^{M\times 1}$ denoting the transmit beamforming vector for user *k*.

The covariance matrix of the transmit signal is computed by

(2) $$R_{X}=E\left[xx^{H}\right]=WW^{H}.$$

Therefore, the transmit power budget can be obtained by

(3) $$\mathrm{Tr}\left[R_{X}\right]\leq P^{\max},$$

where $P^{\max}$ is the transmit power budget.

### 2.1. Communication Model

Let $H_{1}\in C^{N\times M}$ denote the channel matrix from BS to IRS. $h_{k,2}\in C^{N\times 1}$ represents the channel vector from IRS to user *k* with ∀k∈K. The transmitted signal received by the user *k* is given by

(4) $$\begin{array}{cc} y_{c,k}\left(t\right)= & h_{k,2}^{T}\Phi H_{1}x+n_{k}\\ = & h_{k,2}^{T}\Phi H_{1}w_{k}s_{k}+\sum_{i=1,i\neq k}^{K}h_{k,2}^{T}\Phi H_{1}w_{i}s_{i}+n_{k}, \end{array}$$

where $\Phi ≜\mathrm{diag}\left\{\chi_{1}e^{j\phi_{1}},\chi_{2}e^{j\phi_{2}},\cdots ,\chi_{N}e^{j\phi_{N}}\right\}\in C^{N\times N}$ is the diagonal phase shift matrix of the IRS, $\chi_{n}\in \left[0,1\right]$ and $\phi_{n}\in \left[0,2\pi \right)$ indicate the amplitude and phase of element *n* with ∀n∈N, respectively, due to the high overhead of simultaneous implementing of independent control of phase shift and amplitude [13].Therefore, we assume the ideal reflection amplitude of the passive IRS with $\chi_{n}=1,\forall n\in N$ [32]. $n_{k}∼\mathrm{CN}\left(0,\sigma_{c}^{2}\right)$ is the additive white Gaussian noise (AWGN).

We take the Rician fading channel model in this work, and channel $H_{1}$ can be formulated as

(5) $$H_{1}=\sqrt{\frac{\gamma_{1}}{1+\gamma_{1}}}H_{\mathrm{LoS}}+\sqrt{\frac{1}{1+\gamma_{1}}}H_{\mathrm{NLoS}},$$

where $\gamma_{1}$ denotes the Rician factor. $H_{\mathrm{LoS}}\in C^{N\times M}$ and $H_{\mathrm{NLoS}}\in C^{N\times M}$ are LoS component and NLoS component, respectively. The LoS channel matrix can be expanded as $H_{\mathrm{LoS}}=\sqrt{\alpha }e^{j\varphi }a_{r}\left(\theta_{r}\right)b_{t}^{H}\left(\theta_{t}\right)$, where α and φ are the large-scale channel gain and a random phase uniformly distributed in the range from 0 to 2π, respectively. Meanwhile, $a_{r}\left(\theta_{r}\right)\in C^{N\times 1}$ represents the receive steering vector at IRS with the angle of arrival $\theta_{r}$, $b_{t}\left(\theta_{t}\right)\in C^{M\times 1}$ indicates the transmit steering vector of BS with the angle of departure $\theta_{t}$. The steering vector of BS $b\left(\theta \right)$ can be formulated as

(6) $$b\left(\theta \right)=\frac{1}{\sqrt{M}}{\left[1,e^{-j\frac{2\pi }{\lambda }d_{0}\cos\theta },\cdots ,e^{-j\frac{2\pi }{\lambda }\left(M-1\right)d_{0}\cos\theta }\right]}^{T},$$

where $d_{0}$ and λ denote the antennas’ spacing and signal wavelength. Similarly, the steering vector of IRS $a\left(\upsilon ,\theta \right)$ can be formulated as

(7) $$a\left(\upsilon ,\theta \right)=\frac{1}{N}{\left[1,e^{j\frac{2\pi d_{0}}{\lambda }\left(n\;\cos\upsilon \;\cos\theta +n\;\sin\upsilon \;\sin\theta \right)},\cdots ,e^{j\frac{2\pi d_{0}}{\lambda }\left(\left(\sqrt{N}-1\right)\cos\upsilon \;\cos\theta +\left(\sqrt{N}-1\right)\sin\upsilon \;\sin\theta \right)}\right]}^{T}.$$

We leverage the SINR ratio as the performance indicator of communication. Let $\rho_{k}$ denote the SINR of user *k*, which is given by

(8) $$\rho_{c,k}=\frac{{\left|h_{k,2}^{T}\Phi H_{1}w_{k}\right|}^{2}}{\sum_{i=1,i\neq k}^{K}{\left|h_{k,2}^{T}\Phi H_{1}w_{i}\right|}^{2}+\sigma_{c}^{2}}.$$

### 2.2. Radar Sensing Model

At time slot *t*, the received radar echo signal at BS can be expressed as

(9) $$\begin{array}{c} y_{r}\left(t\right)=H_{1}^{H}\Phi A\Phi^{H}H_{1}\times \left(t-\tau_{k}\right)+n_{r}\left(t\right) \end{array}$$

where $A\in C^{N\times N}$ represents the target response matrix of IRS. $\tau_{k}$ denotes the propagation delay between the transmitter and the target. The $n_{r}\left(t\right)$ is AWGN with $n_{r}\left(t\right)∼\mathrm{CN}\left(0,\sigma_{r}^{2}I_{M}\right)$. The specific formulas are listed as

(10) $$A=\sum_{k=1}^{K}\beta_{k}a\left(\upsilon_{k},\theta_{k}\right)a^{H}\left(\upsilon_{k},\theta_{k}\right).$$

The received sensing echo signal from the *k*-th target $y_{r,k}\in C^{M\times 1}$ can be formulated as

(11) $$y_{r,k}=H_{1}^{H}\Phi A\Phi^{H}H_{1}w_{k}s_{k}\left(t-\tau_{k}\right)+\sum_{k^{\prime }\in K\setminus \left\{k\right\}}H_{1}^{H}\Phi A\Phi^{H}H_{1}w_{k^{\prime }}s_{k^{\prime }}\left(t-\tau_{k^{\prime }}\right)+n\left(t\right).$$

We use the SINR as the sensing performance indicator [33]. Therefore, the SINR of the radar can be given by

(12) $$\rho_{r,k}=\frac{{∥H_{1}^{H}\Phi A\Phi^{H}H_{1}w_{k}∥}_{F}^{2}}{\sum_{k^{\prime }\in K\setminus \left\{k\right\}}{∥H_{1}^{H}\Phi A\Phi^{H}H_{1}w_{k^{\prime }}∥}_{F}^{2}+M\sigma_{r}^{2}},$$

### 2.3. Computation Offloading Model

The UE generates a series of data processing tasks that need to be executed in a timely manner for the low latency requirement. Due to the constrained energy and computation resources of UE, the task can be offloaded to the BS. The computation task generated by UE $k\left(k\in K\right)$ at time slot *t* is denoted by a tuple $D_{k}\left(t\right)=\left\{d_{k}\left(t\right),c_{k}\left(t\right),\xi_{k}\left(t\right)\right\}$, where $d_{k}\left(t\right)$ denotes the input data size (bits), $c_{k}\left(t\right)$ represents the required computation cost (e.g., the number of CPU cycles for processing one-bit data), and $\xi_{k}\left(t\right)$ indicates the maximum tolerable latency of UE *k*, respectively. We assume that tasks are bitwise separable and can be partially executed locally, while the remaining parts directly send the raw data to the BS for processing. The processing delay of the BS server executing task $D_{k}\left(t\right)$ can be calculated by

(13) $$T_{o,k}=\frac{d_{k}\left(t\right)c_{k}\left(t\right)}{f_{o,k}\left(t\right)},$$

where the $f_{o,k}\left(t\right)$ represents the CPU frequency of the BS server. The processing delay of UE *k* to execute the task $D_{k}\left(t\right)$ locally can be written as

(14) $$T_{l,k}=\frac{d_{k}\left(t\right)c_{k}\left(t\right)}{f_{l,k}\left(t\right)},$$

where $f_{l,k}$ is the CPU frequency of UE *k* (cycles/s). The overall latency for processing the task $D_{k}\left(t\right)$ is depicted as

(15) $$T_{k}^{\mathrm{tol}}=w_{k}\left(T_{o,k}+T_{u,k}+T_{d,k}\right)+\left(1-w_{k}\right)T_{l,k},$$

where $w_{k}\in \left[0,1\right]$ represents the offloading ratio. Under two extremes, $w_{k}=1$ when the task is offloaded to BS and $w_{k}=0$ when the task is processed locally at the UE *k*. $T_{u,k}=d_{k}\left(t\right)/r_{k}\left(t\right)$ is the uplink transmission delay with the uplink data rate $r_{k}$, which is listed as

(16) $$r_{k}\left(t\right)=B_{k}\log_{2}\left(1+\frac{p_{k}{\left|h_{m,1}^{T}\Phi h_{k,2}\right|}^{2}}{\sum_{i=1,i\neq k}^{K}p_{i}{\left|h_{m,1}^{T}\Phi h_{i,2}\right|}^{2}+\sigma_{c}^{2}}\right),$$

where $B_{k}$ and $p_{k}$ are the uplink transmit bandwidth and power for UE *k*, respectively. Due to the small size of the processing result, the latency of receiving the result $T_{d,k}$ can be ignored [34,35].

Meanwhile, the energy consumption of executing task offloading by the UE *k* can be denoted by

(17) $$E_{o,k}=\kappa_{o}f_{o,k}^{2}\left(t\right)d_{k}\left(t\right)c_{k}\left(t\right),$$

where $\kappa_{o}$ denotes the effective capacitance coefficients related to the chip architecture [36,37]. The energy consumption for UE *k* executing the task locally can be formulated as

(18) $$E_{l,k}=\kappa_{l}f_{l,k}^{2}\left(t\right)d_{k}\left(t\right)c_{k}\left(t\right).$$

Similarly, $\kappa_{l}$ is the effective capacitance coefficient. Therefore, the overall energy consumption can be given by

(19) $$E_{k}^{\mathrm{tol}}=w_{k}\left(E_{o,k}+E_{u,k}+E_{d,k}\right)+\left(1-w_{k}\right)E_{l,k},$$

where $E_{u,k}$ represents the offloading energy consumption with $E_{u,k}=p_{k}d_{k}/r_{k}$. The energy consumption for result receiving can also be ignored.

## 3. Problem Formulation

This section studies the performance optimization and trade-offs of sensing, data transmission, and computation offloading. The overall system performance is optimized through joint beamforming, phase shifting design, and resource allocation.

### 3.1. Transmission Performance Optimization

The optimization goal of the IRS-assisted ISAC system is to maximize the data rate while satisfying the sensing performance requirement. Then, the objective of data transmission optimization can be formulated as follows:

(20) $$\max_{W,\Phi }\;\Psi_{1}=\sum_{k=1}^{K}\log_{2}\left(1+\rho_{c,k}\right),$$

subject to

(21) $$\mathrm{Tr}\left[R_{X}\right]\leq P^{\max},$$

(22) $$R_{X}⪰0,$$

(23) $$\rho_{r,k}\geq \rho^{\mathrm{thr}},\forall k\in K,$$

where $\rho^{\mathrm{thr}}$ is a threshold value for the radar SINR. Constraint (21) depicts the transmit power limit for deploying the ISAC. Constraint (23) ensures the sensing performance while optimizing the communication performance.

### 3.2. System Energy Consumption Optimization

Due to the strained resources of UE, it is necessary to optimize UE energy consumption. The optimization objective for computation offloading is to minimize system energy consumption for the system while satisfying the latency constraints, which is written as

(24) $$\min_{f_{o,k},f_{l,k}}\;\Psi_{2}=\sum_{k=1}^{K}E_{k}^{\mathrm{tol}},$$

subject to

(25) $$\sum_{k=1}^{K}f_{o,k}\leq F_{o}^{\mathrm{tol}},$$

(26) $$f_{l,k}\leq f_{l,k}^{\mathrm{tol}},\forall k\in K,$$

(27) $$T_{k}^{\mathrm{tol}}\leq \xi_{k}\left(t\right),\forall k\in K,$$

(28) $$w_{k}\in \left[0,1\right],\forall k\in K,$$

where $F_{o}^{\mathrm{tol}}$ and $f_{l,k}^{\mathrm{tol}}$ indicate the total computing resource of BS server and local computing resource of UE *k*, respectively. Constraints (25)–(27) represent the computing resource limitation of BS, maximum local computation resource, and latency constraint for processing the task $D_{k}\left(t\right)$. Constraint (28) represents the offloading decision.

### 3.3. System-Comprehensive Performance Optimization

In this work, we aim to optimize the system’s transmission performance and energy consumption through joint beamforming, phase design, and power allocation. Considering that there is a coupling relationship between optimization objects (21) and (23), we can reformulate the optimization problem as

(29) $$\max_{W,\Phi ,f_{o,k},f_{l,k}}\left(\Psi_{1},-\Psi_{2}\right),$$

subject to (21)–(23), (25)–(28).

The downlink sum data rate is related to the number of users, transmit power, and sensing requirement of quality, which can be maximized through reasonable beamforming and phase shift design. Meanwhile, the total energy consumption of the system can be optimized by appropriate computation offloading decisions. The optimization problem (29) is NP-hard and non-convex; thus, using mathematical methods to solve it will bring substantial computational complexity. Moreover, considering the time-varying wireless channel environment, a model-free DRL approach is adopted to obtain the optimal solution.

## 4. DRL-Based Joint Task Offloading and Resource Allocation Scheme

In this section, we formulate the optimization goal as an MDP. Then, we propose two improved DRL-based schemes to solve the joint precoding and computation offloading problem in the IRS-aided ISAC system.

### 4.1. MDP Formulation

We use a four-elements tuple $\left(S,A,P,R\right)$ to denote the MDP, where S and A denote the set of system state and actions, respectively. P is the state transition probability and R represents the reward function. We can outline the process of RL interacting with the environment as follows. The agent adopts action $a_{t}$ under environment state $s_{t}$, and receives the instant reward $r_{t}$ as the response for the action $a_{t}$. Then, the environment state $s_{t}$ turns to new $s_{t}$ according to the transition function $P(s_{t},a_{t},s_{t+1})$. The reinforcement learning aims to obtain the optimal policy $\pi^{*}\left(a|s\right)$ from a given MDP, which is the mapping from state to action that can obtain the maximum long-term cumulative reward $R_{t}=\sum_{i=0}^{\infty }\gamma^{i}R\left(s_{t+i+1},a_{t+i+1}\right)$. γ∈[0,1) is the discount factor. We can define state, action, and reward in our model as follows.

State: The environmental state at the *t*-th time step consists of channel matrices, BS transmit power, the size of the computation task, and the action adopted by the agent in (t−1)-th time step. Thus, the state of agent $s_{t}\in S$ is given by

(30) $$s_{t}=\left\{{\bar{H}}_{1}\left(t\right),{\bar{H}}_{2}\left(t\right),p\left(t\right),d\left(t\right),a\left(t-1\right)\right\},$$

where

- H¯1t=ReH1t,ImH1t: the channel matrix $H_{1}\left(t\right)$ is divided into the real part and imaginary part, due to the fact that the neural network cannot deal with the complex value.
- H¯2t=ReH2t,ImH2t: as the same way, $H_{2}\left(t\right)$ is separated into two independent parts, and $H_{2}\left(t\right)=\left\{h_{k,2}\left(t\right)|k\in K\right\}$.
- p(t)=Repkt,Impkt∣∀k∈K: the transmit power for each UE and divided into two ports inputting the training network with $p_{k}\left(t\right)=\mathrm{Tr}\left(w_{k}w_{k}^{H}\right)$.
- $d\left(t\right)=\left[d_{k}\left(t\right)|\forall k\in K\right]$: the size of the computation task generated at UE.
- a(t−1): denotes the action selected by the agent at the previous time step.

Action: The action of the agent comprises the transmit beamforming matrix at BS, phase shift of IRS, and computation offloading decision. We can formulated the action $a_{t}\in A$ as

(31) $$a_{t}=\left\{\bar{W}\left(t\right),\Phi \left(t\right),w_{k}\left(t\right)\right\},$$

where W¯(t)=ReWt,ImWt and Φ¯t=ReΦt,ImΦt indicate the real and imaginary parts of transmit beamforming and phase shift matrices. $w_{k}\in \left[0,1\right]$ for ∀k∈K is the computation offloading action.

Reward: The agent, through the feedback of reward, evaluates the action and makes improvements. This work aims to optimize the communication data rate while minimizing the system energy consumption. Thus, the reward $r_{t}$ at the *t*-th time step is defined by

(32) $$r_{t}=\omega_{1}\Psi_{1}\left(t\right)-\omega_{2}\Psi_{2}\left(t\right),$$

where $\omega_{1}$ and $\omega_{2}$ are the weighting factors with $\omega_{1}+\omega_{2}=1$. The weighting factor can be used for control optimization preferences.

### 4.2. An Improved DDPG-Based Joint Optimization Algorithm

**Figure 2:**
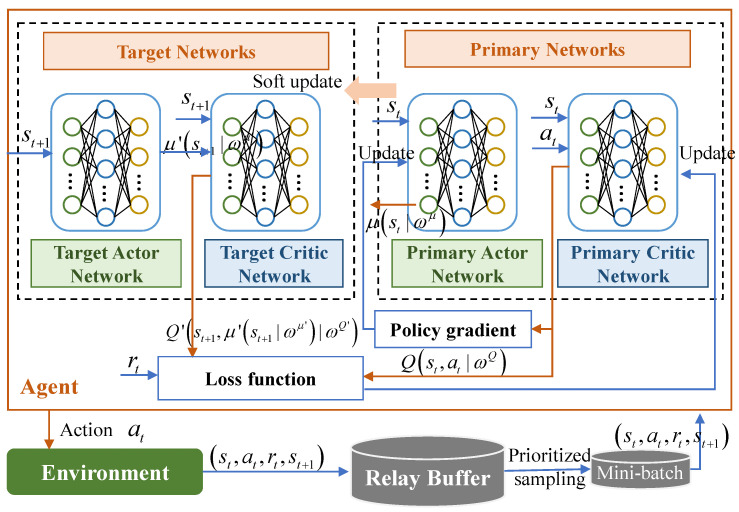
Proposed task offloading and resource allocation framework based on DDPG.

Considering that the transmit power, phase shift, and the offloading scale factor are continuous variables, we are resorting to the policy-based scheme. The DDPG algorithm has been proven as an effective solution for the continuous control problem [23]. Thus, the DDPG-based scheme is developed in this work. Figure 2 depicts the developed framework. The proposed DRL model adopted the evaluate network and target network with identical structures but differing parameters. Both evaluate and target networks contain a set of actor-critic neural networks.

At each time slot, the evaluate network obtains environmental state $s_{t}$ and then outputs the action $a_{t}$. The *Q* value is adopted to describe the long-term reward of executing $a_{t}$, which can be calculated by the Bellman equation [38]

(33) $$Q^{\mu }\left(s_{t},a_{t}\right)=E\left[r_{t}\left(s_{t},a_{t}\right)+\gamma Q^{\mu }\left(s_{t+1},a_{t+1}\right)\right],$$

where μ:S←A is the deterministic policy function, and the actor function $\mu \left(s|\omega^{\mu }\right)$ works by mapping a state to an action to specific current policy. The DRL agent interacts with the environment to find the optimal action corresponding to the maximum *Q* value

(34) $$Q^{*}\left(s_{t},a_{t}\right)=E\left[r_{t}\left(s_{t},a_{t}\right)+\gamma \max_{a_{t+1}}\;Q^{*}\left(s_{t+1},a_{t+1}\right)\right].$$

The experience replay mechanism is leveraged to break the correlation between experience tuples [39]. Applying *J* tuples sampled from the experience buffer, the critic network is trained by minimizing the loss function

(35) $$L\left(\omega^{Q}\right)=\frac{1}{J}\sum_{i\in J}{\left(y_{i}-Q\left(s_{i},a_{i}|\omega^{Q}\right)\right)}^{2},$$

where

(36) $$y_{i}=r\left(s_{i},a_{i}\right)+\gamma Q^{\prime }\left(s_{i+1},a_{i+1}|\omega^{Q^{\prime }}\right),$$

denotes the target value. $\omega^{Q^{\prime }}$ represents the parameters of the function approximator.

The actor network updating following the policy gradient rule and the loss function can be expressed as

(37) $$\nabla_{\omega^{\mu }}J=\frac{1}{J}\sum_{i\in J}\left[\nabla_{a}Q\left(s,a|\omega^{Q}\right)\left|s=s_{i},a=\mu \left(s_{i}\right)\nabla_{\omega^{\mu }}\mu \left(s|\omega^{\mu }\right)\right|s=s_{i}\right].$$

To address the unstable issue in the learning process, the soft target is leveraged for the updating of target actor-critic networks, which can be formulated by

(38) $$\omega^{\mu^{\prime }}\leftarrow \tau \omega^{\mu }+\left(1-\tau \right)\omega^{\mu^{\prime }},$$

(39) $$\omega^{Q^{\prime }}\leftarrow \tau \omega^{Q}+\left(1-\tau \right)\omega^{\mu^{\prime }},$$

with the soft update factor τ≪0.

The experience replay mechanism overcomes the problem prone to divergence in the training process. Since the conventional experience replay mechanism replayed the transition tuples uniformly, the importance of different experiences is ignored. The prioritized experience replay (PER) assigns priorities based on the importance of the experience samples, which is adopted to speed up the training convergence [39]. The internal logic of the PER mechanism is to replay extremely good or bad experiences more frequently. The temporal difference error (TD-error) is usually leveraged as the measurement of the experiences’ value [40]. The absolute TD-error is proportional to the correction to the expected action value. The TD-error of transition tuple *i* can be formulated by

(40) $$\delta_{i}=y_{i}-Q\left(s_{i},a_{i}|\omega^{Q}\right).$$

The probability of the transition *i* is given by

(41) $$P\left(i\right)=\frac{p_{i}^{\varrho }}{\sum_{k}p_{k}^{\varrho }},$$

where $\frac{1}{\mathrm{rank}\left(i\right)}$, $\mathrm{rank}\left(i\right)$ represents the ranking of transition *i* when sorted according to the absolute TD-error. ϱ is the degree of priority adopted. However, PER changes the state access frequency, introduces the bias, and may cause oscillation and divergence. Thus, the importance-sampling weights are employed to handle the bias with $W_{i}=1/{\left[B\cdot P\left(i\right)\right]}^{\beta }$, B denotes replay buffer size, and β is the factor that controls the degree of correction. The proposed DRL-based joint task offloading and resource allocation algorithm is summarized in Algorithm 1.   
**Algorithm 1** PER DDPG-based Joint Task Offloading and Resource Allocation Algorithm.**Input**: $H_{1}$, $h_{k,2}$, $\mu \left(s|\omega^{\mu }\right)$, $Q\left(s,a|\omega^{Q}\right)$, learning rates $\alpha_{\mu }$ and $\alpha_{Q}$, τ, γ**Output**: W, Φ, $w_{k}$1: Initialize actor parameter $\omega^{\mu }$, critic network parameter $\omega^{Q}$, target actor network parameter with $\omega^{\mu^{\prime }}\leftarrow \;\omega^{\mu }\;$, critic network parameter with $\omega^{Q^{\prime }}\leftarrow \;\omega^{Q}\;$, replay buffer with size B, minibatch *J*2: Initialize transmit beamforming matrix W, phase shift matrix Φ3: **For** episode=0,1,2,⋯,E−1 **do**4:    Initialize random noise $n_{e}$ for the action exploration5:    Initialize environment state $s_{0}$6:    **For** time step t=0,1,2,⋯,T−1 **do**7:        Select action $a_{t}$ based on (31) and noise $n_{e}$8:        Execute action $a_{t}$, calculate instant reward $r_{t}$ and turn to next state $s_{t+1}$9:        Record the tuple $\left(s_{t},a_{t},r_{t},s_{t+1}\right)$ into the replay buffer10:       **If** t>B **then**11:            **For** i=0,1,2,⋯,J−1 **do**12:                Sample tuple *i* according to probability P(i)13:                Compute the importance-sampling weights $W_{i}$ and TD-error $\delta_{i}$14:                Update the priority of tuple *i*15:            **End for**16:            Update the critic network parameter by minimize the loss (35)17:            Update the actor network parameter with policy gradient (37)18:            Update target actor and critic parameters according to (38) and (39)19:       **End if**20:    **End for**21: **End for**

### 4.3. Twin Delayed DDPG (TD3)-Based Joint Optimization Algorithm

**Figure 3:**
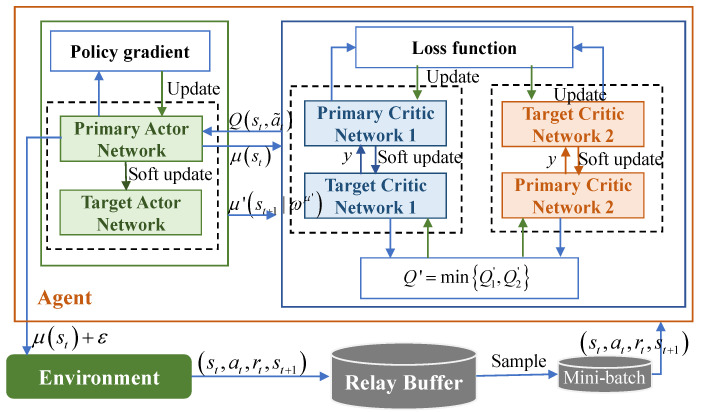
Proposed task offloading and resource allocation framework based on TD3.

The TD3 algorithm is considered as an improver of DDPG, which solves a series of issues caused by overestimation in the process of the *Q* value estimate in DDPG [41]. We depicts the TD3-based joint optimization framework in Figure 3. Although the overestimated values are small in each update, they may accumulate after every update, creating a significant bias. Furthermore, the inaccurate *Q* value leads to the deterioration of the policy network. This process forms a feedback loop in which suboptimal behavior is continuously reinforced. The TD3 algorithm addresses the above-mentioned challenge through the following technologies.

Firstly, clipped double *Q* learning. The TD3 leverages twin critic networks to estimate two *Q* function, and choose the smaller one as the target *Q* value to compute loss in the Bellman equation. The target update in the double critic networks framework is formulated as

(42) $$y_{i}=r\left(s_{i},a_{i}\right)+\gamma \min_{n=1,2}Q_{n}^{\prime }\left(s_{i+1},a_{i+1}|\omega^{Q_{n}^{\prime }}\right),$$

where $\omega^{Q_{n}^{\prime }}\left(n=1,2\right)$ denote weight parameters of two target critic networks, respectively. Critic networks are updated by using the loss function, which are given by

(43) $$L\left(\omega^{Q_{1}}\right)=\frac{1}{J}\sum_{i\in J}{\left(y_{i}-Q_{1}\left(s_{i},a_{i}|\omega^{Q_{1}}\right)\right)}^{2},$$

(44) $$L\left(\omega^{Q_{2}}\right)=\frac{1}{J}\sum_{i\in J}{\left(y_{i}-Q_{2}\left(s_{i},a_{i}|\omega^{Q_{2}}\right)\right)}^{2},$$

where $\omega^{Q_{1}}$ and $\omega^{Q_{2}}$ indicate weight parameters of two estimate critic networks, respectively. The smaller value is adopted for the Bellman error function. Secondly, delayed policy updates. The actor and its target network reduce the update frequency compared to critic networks, to avoid the divergent behavior caused by the policy updates under inaccurate value estimate. Thirdly, target policy smoothing. A regularization strategy is leveraged in TD3 to address the overfit at high peaks and *Q* value error. In practice, a random noise is added in the action selection process to enforce the generalization of similar actions as given by

(45) $${\tilde{a}}_{t+1}\leftarrow \mu \left(s_{t+1}|\omega^{\mu }\right)+\epsilon ,$$

where the added noise $\epsilon ∼clip\left(N\left(0,\sigma_{a}\right),-c,c\right)$ is clipped by the constant *c* to ensure the proximity between the target action and the original. The TD3-based joint optimization algorithm is similar to the processing process of Algorithm 1, with improvements in the following aspects:

- In the Input Step, input two pairs of critic networks $Q_{1}\left(s,a|\omega^{Q_{1}}\right)$ and $Q_{2}\left(s,a|\omega^{Q_{2}}\right)$, respectively. In Step 1, initialize parameters of two estimate critics and two target critics with $\omega^{Q_{1}}$, $\omega^{Q_{2}}$, $\omega^{Q_{1}^{\prime }}$, and $\omega^{Q_{2}^{\prime }}$.
- Target policy smoothing is realized by (45). Then, the agent updates the target value using (42). In Step 16, the loss is computed by (43) and (44).
- Before turning to Step 17, the agent adopts a delayed update strategy to keep policy networks updated less frequently than value networks.

## 5. Numerical Results

In this section, the simulation results are presented to assess the proposed DRL-based task offloading and resource allocation schemes in the IRS-assisted ISAC system. The simulation is based on Python 3.8 and PyTorch 1.8.0. We assume that the BS and the IRS are located at $\left[-10,0,0\right]$ m and $\left[90,0,2\right]$ m. UEs are randomly distributed in a radius of 1 m below the IRS [21]. The channel matrix $H_{1}$ and $h_{k,2}$ with k∈K follow the Rician distribution with the Rician factor $\gamma_{1}=3$ dB [42]. According to [43], the carrier frequency is set to 30 GHz, and the shadowing standard deviation is 7.8 dB. The path-loss exponent of BS-IRS and IRS-UE are set to 2.8 and 2.5 [44]. The noise power $\sigma^{2}=-174$ dBm/Hz. Meanwhile, we set noise power $\sigma^{2}=-85$ dBm and the bandwidth $B_{k}=2$ MHz [43]. The input data size of task $d_{k}$, required computation cost $c_{k}$, and CPU frequency of BS server $f_{l,k}$ are randomly generated in the interval [1, 2] Mbits, [1, 3] Kcycles/bit, and [1, 2] Gcycles/s, respectively [45]. CPU frequency of BS server $F_{o}^{\mathrm{tol}}$, effective capacitance coefficients $\kappa_{o}$, and $\kappa_{l}$ are set to 10 Gcycles/s, $10^{-26}$, and $3\times 10^{-26}$ [45]. The default simulation parameter is listed in Table 2.

### 5.1. Convergence Performance

**Figure 4:**
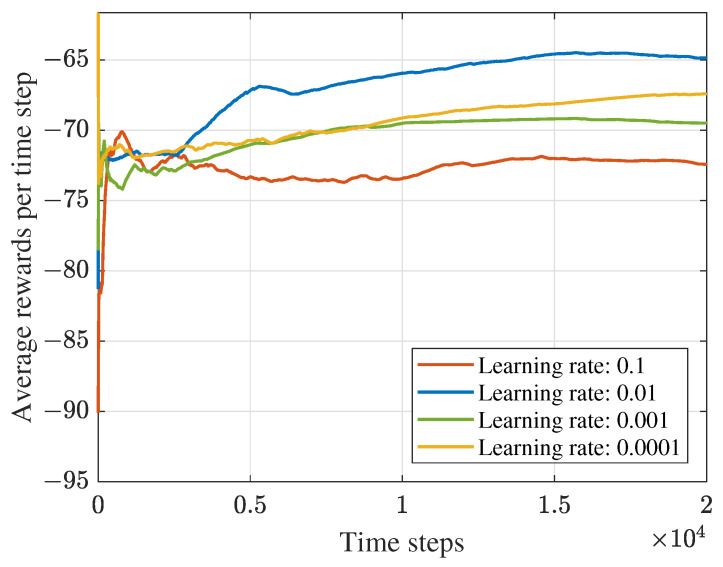
Convergence performance under different learning rates.

**Figure 5:**
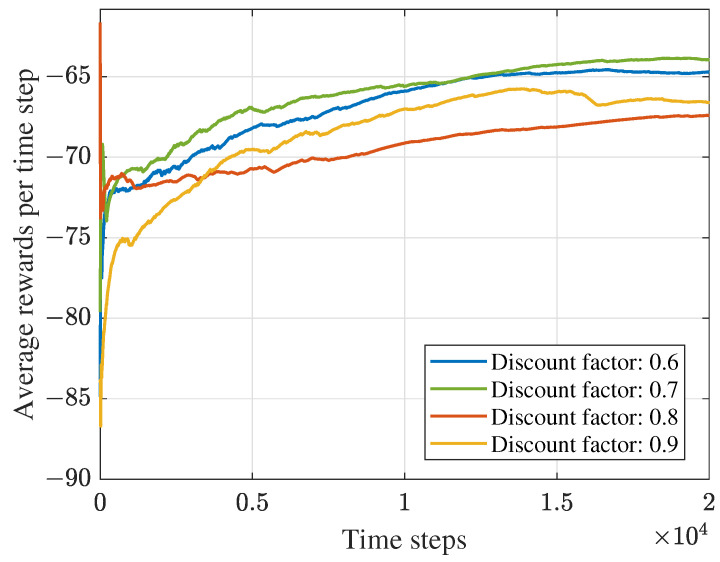
Convergence performance under different discount factors.

Considering the relationship between the DDPG-based algorithm’s performance and the parameters in the system, we first conducted several experiments to find the appropriate learning rate and discount factor. Meanwhile, as shown in Figure 4 and Figure 5, the proposed DDPG-based algorithm’s convergence performance is displayed. Figure 4 depicts the average rewards under different learning rates. The average reward is obtained by $\sum_{t=1}^{T_{i}}\frac{r_{t}}{N_{i}}\left(N_{i}=1,2,\cdots ,T_{\max}\right)$, where $T_{\max}$ denotes the maximum time steps. It can be obtained from the figure that the maximum average reward can be achieved when the learning rate is 0.01. Figure 5 is the convergence performance under different discount factors. The figure shows that the algorithm performs better than others when the discount value is 0.7. Therefore, we set the learning rate and discount value as 0.01 and 0.7 in the following experiments for the DDPG-based framework. Moreover, it can be obtained that average rewards increase with the number of training time steps and finally converge at about $10^{4}$ rounds.

### 5.2. Performance Comparison

**Figure 6:**
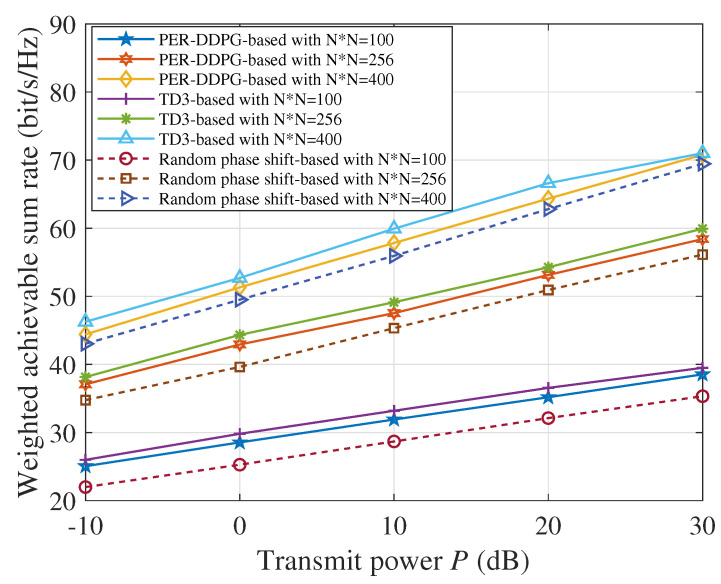
The weighted achievable data rate versus the transmit power budget.

We compare the performance of the PER-DDPG-based scheme, TD3-based scheme, and random IRS phase scheme under different transmit power budgets and different numbers of IRS elements. We set the number of IRS elements N×N as 100, 256, and 400, and the number of users *K* as 10, 16, and 20, respectively. Figure 6 illustrates that the achievable weighted communication data rate is directly proportional to the maximum transmit power budget and the number of IRS elements. It can be seen from the figure that the TD3-based algorithm achieves the best communication performance, the PER-DDPG-based algorithm is slightly inferior to the TD3 scheme, and the random phase shift-based one has the worst performance.

**Figure 7:**
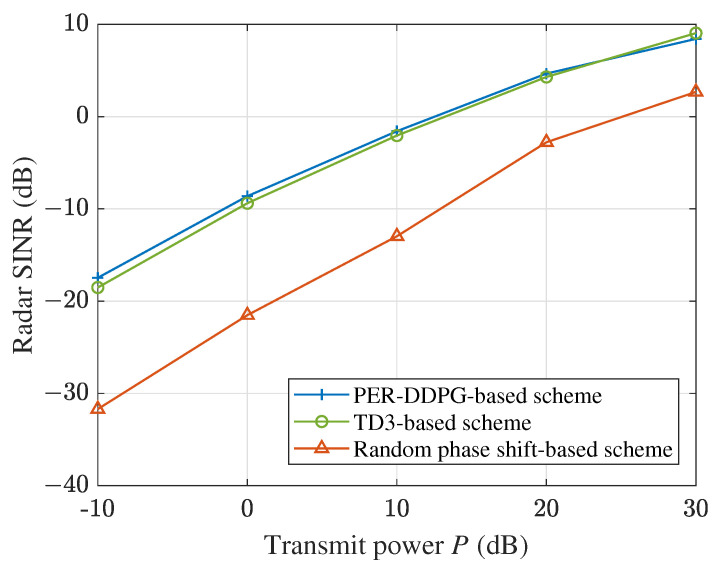
Sensing SINR versus the transmit power budget.

Figure 7 plots the sensing SINR versus the transmit power budget, where the number of users *K* and IRS’s elements N×N are set to 10 and 100, respectively. The radar sensing SINR consistently increases with the expansion of transmit power budget, but the growth speed gradually slows down. Our proposed DRL-based algorithms show a better performance than the baseline.

**Figure 8:**
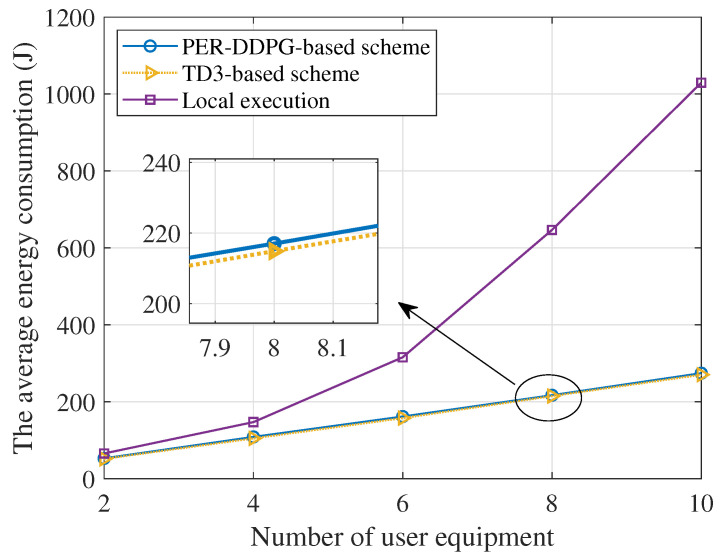
The total energy consumption versus the number of users.

Figure 8 depicts the correlation between the number of users and the system energy consumption. We set the number of IRS elements N×N as 100. As depicted in the plot, it is evident that the system energy consumption increases with the growing number of users. With the rising number of users, the amount of tasks offloaded to the base station rises, resulting in a growth in energy consumption. The proposed two schemes dramatically reduce the overall execution energy consumption compared with local execution methods, and the TD3-based scheme is slightly better than the PER-DDPG-based scheme.

**Figure 9:**
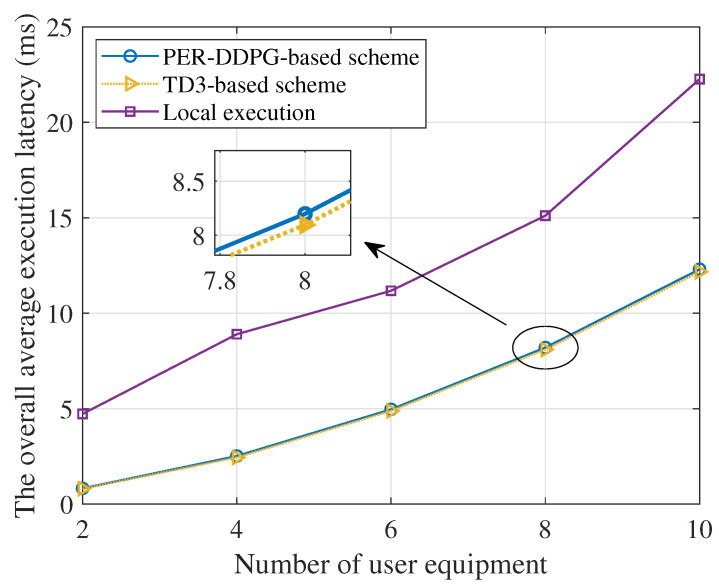
The total average execution latency versus the number of users.

Figure 9 describes the relationship between the number of users and the offloading delay, and the number of IRS elements N×N is set to 100. The figure demonstrates that an increase in the number of users leads to a rise in data processing time due to resource competition among the users. Compared with the local execution method, the proposed DRL methods greatly reduce the overall average execution delay of the task, and the TD3 algorithm has the lowest total delay.

## 6. Conclusions

In this paper, we studied the IRS-assisted ISAC framework, wherein the IRS is exploited to establish virtual links in NLoS areas for enhancing radar sensing performance and communication data rate. We aim to improve the system’s transmission and energy efficiency through joint task offloading and resource allocation under constraints of transmit power budget, sensing quality, and tolerable latency of offloading. Specifically, transmit beamforming, IRS phase shift, and task offloading are jointly designed, and the weight coefficient is leveraged to control the balance between performance and overhead. The PER DDPG-based and TD3-based algorithms are developed for the complex optimization problem. Numerical results demonstrate that the proposed algorithms have better performance than the baseline scheme. In addition, the simulation shows that the system performance is related to the transmit power, the number of IRS components, and the number of users. In practical applications, we can optimize system performance by setting parameters reasonably. In future work, we will combine the distributed DRL algorithm and federated learning framework to improve the efficiency and scalability of the joint optimization scheme in large-scale networks. Meanwhile, extended to multi-IRS scenarios, our proposed method suffers from the action space explosion problem caused by the exponential increase in intermediate channel coefficients. Therefore, the meta-reinforcement learning can be adopted to decompose the cascaded channel, and reduce the solution complexity and computational overhead. Moreover, future experiments will focus on implementing and testing the proposed strategies in real environments, striving to translate the theoretical potential into practical gains.

## Figures and Tables

**Table 1:** Comparison with the state of the art.

Ref.	Phases	Users	Targets	Radar Paths	Method
[13]	Continuous	Single	Single	LoS, NLoS	MM
[8]	Continuous	Single	Multiple	NLoS	SDR
[15]	Continuous	Multiple	Multiple	LoS	AO
[18]	Continuous	Single	Single	LoS, NLoS	PDD, BCD
[19]	Discrete	Multiple	Multiple	LoS	AO
[20]	Continuous	Multiple	Single	LoS, NLoS	ADMM, AO
[21]	Discrete	Multiple	Multiple	NLoS	SDR
[22]	Continuous	Single	Multiple	LoS, NLoS	BCD
This paper	Continuous	Multiple	Multiple	NLoS	DRL

**Table 2:** Parameter values.

Parameter	Description	Value
*M*	Number of antennas at BS	8
N×N	Number of IRS elements	64
*K*	Number of UEs	8
$P^{\max}$	Power budget of BS	10 dB
$p_{k}$	Transmit power of the UE	30 dBm
$\sigma^{2}$	Noise variance	−85 dBm
$B_{k}$	Bandwidth allocated to UE *k*	2 MHz
$d_{k}$	Input data size of task	U[1,2] Mbits
$c_{k}$	Required computation cost	U[1,2] Kcycles/bit
$F_{o}^{\mathrm{tol}}$	CPU frequency of BS server	10 Gcycles/s
$f_{l,k}$	CPU frequency of UE	U[1,2] Gcycles/s
$\xi_{k}$	Maximum tolerable latency	100 ms
$\kappa_{o},\kappa_{l}$	Effective capacitance coefficient	$10^{-26}$, $3\times 10^{-26}$
$\alpha_{\mu },\alpha_{Q}$	Learning rate for actor and critic networks	0.001, 0.001
γ	Discount factor	0.7
ϵ	Soft update factor	0.01
M	Capacity of experience buffer	10,000
*J*	Capacity of minibatch	16

## Data Availability

The datasets used in this paper available from the corresponding author upon request.

## References

[1] (2023). ITU-R WP5D. Draft New Recommendation ITU-R M. [IMT. Framework for 2030 and Beyond]–Framework and Overall Objectives of the Future Development of IMT for 2030 and Beyond. https://www.itu.int/md/R19-WP5D-230612-TD-0905/.

[2] Mishra K.V., Shankar M.B., Koivunen V., Ottersten B., Vorobyov S.A. (2019). Toward millimeter-wave joint radar communications: A signal processing perspective. IEEE Signal Process. Mag..

[3] Kumari P., Vorobyov S.A., Heath R.W. (2019). Adaptive virtual waveform design for millimeter-wave joint communication–Radar. IEEE Trans. Signal Process..

[4] Dokhanchi S.H., Mysore B.S., Mishra K.V., Ottersten B. (2019). A mmWave automotive joint radar-communications system. IEEE Trans Aerosp. Electron. Syst..

[5] Zhang Q., Sun H., Gao X., Wang X., Feng Z. (2022). Time-Division ISAC Enabled Connected Automated Vehicles Cooperation Algorithm Design and Performance Evaluation. IEEE J. Sel. Areas Commun..

[6] Liu X., Zhang H., Long K., Zhou M., Li Y., Poor H.V. (2022). Proximal Policy Optimization-Based Transmit Beamforming and Phase-Shift Design in an IRS-Aided ISAC System for the THz Band. IEEE J. Sel. Areas Commun..

[7] Solomitckii D., Heino M., Buddappagari S., Hein M.A., Valkama M. (2021). Radar scheme with raised reflector for NLOS vehicle detection. IEEE Trans. Intell. Transp. Syst..

[8] Song X., Zhao D., Hua H., Han T.X., Yang X., Xu J. Joint transmit and reflective beamforming for IRS-assisted integrated sensing and communication. Proceedings of the 2022 IEEE Wireless Communications and Networking Conference (WCNC).

[9] Liu F., Cui Y., Masouros C., Xu J., Han T.X., Eldar Y.C., Buzzi S. (2022). Integrated sensing and communications: Toward dual-functional wireless networks for 6G and beyond. IEEE J. Sel. Areas Commun..

[10] Rajatheva N., Atzeni I., Björnson E., Bourdoux A., Buzzi S., Doré J.B., Erkucuk S., Fuentes M., Guan K., Hu Y. (2020). White paper on broadband connectivity in 6G. http://urn.fi/urn:isbn:9789526226798.

[11] Shao X., You C., Ma W., Chen X., Zhang R. (2022). Target sensing with intelligent reflecting surface: Architecture and performance. IEEE J. Sel. Areas Commun..

[12] Liu X., Huang T., Shlezinger N., Liu Y., Zhou J., Eldar Y.C. (2020). Joint transmit beamforming for multiuser MIMO communications and MIMO radar. IEEE Trans. Signal Process..

[13] Jiang Z.M., Rihan M., Zhang P., Huang L., Deng Q., Zhang J., Mohamed E.M. (2022). Intelligent Reflecting Surface Aided Dual-Function Radar and Communication System. IEEE Syst. J..

[14] Chu Z., Xiao P., Shojafar M., Mi D., Mao J., Hao W. (2021). Intelligent Reflecting Surface Assisted Mobile Edge Computing for Internet of Things. IEEE Wirel. Commun. Lett..

[15] Sankar R.P., Chepuri S.P. Beamforming in Hybrid RIS assisted Integrated Sensing and Communication Systems. Proceedings of the 2022 30th European Signal Processing Conference (EUSIPCO).

[16] Buzzi S., Grossi E., Lops M., Venturino L. (2022). Foundations of MIMO Radar Detection Aided by Reconfigurable Intelligent Surfaces. IEEE Trans. Signal Process..

[17] Hua M., Wu Q., He C., Ma S., Chen W. (2023). Joint Active and Passive Beamforming Design for IRS-Aided Radar-Communication. IEEE Trans. Wirel. Commun..

[18] He Y., Cai Y., Mao H., Yu G. (2022). RIS-Assisted Communication Radar Coexistence: Joint Beamforming Design and Analysis. IEEE J. Sel. Areas Commun..

[19] Wang X., Fei Z., Huang J., Yu H. (2022). Joint Waveform and Discrete Phase Shift Design for RIS-Assisted Integrated Sensing and Communication System Under Cramer-Rao Bound Constraint. IEEE Trans. Veh. Technol..

[20] Liu R., Li M., Liu Y., Wu Q., Liu Q. (2022). Joint Transmit Waveform and Passive Beamforming Design for RIS-Aided DFRC Systems. IEEE J. Sel. Top. Signal Process..

[21] Liao C., Wang F., Lau V.K.N. (2023). Optimized Design for IRS-Assisted Integrated Sensing and Communication Systems in Clutter Environments. IEEE Trans. Commun..

[22] Huang N., Wang T., Wu Y., Wu Q., Quek T.Q.S. (2022). Integrated Sensing and Communication Assisted Mobile Edge Computing: An Energy-Efficient Design via Intelligent Reflecting Surface. IEEE Wirel. Commun. Lett..

[23] Lillicrap T.P., Hunt J.J., Pritzel A., Heess N., Erez T., Tassa Y., Silver D., Wierstra D. (2015). Continuous control with deep reinforcement learning. arXiv.

[24] LeCun Y., Bengio Y., Hinton G. (2015). Deep learning. Nature.

[25] François-Lavet V., Henderson P., Islam R., Bellemare M.G., Pineau J. (2018). An introduction to deep reinforcement learning. Found. Trends Mach. Learn..

[26] Mnih V., Kavukcuoglu K., Silver D., Rusu A.A., Veness J., Bellemare M.G., Graves A., Riedmiller M., Fidjeland A.K., Ostrovski G. (2015). Human-level control through deep reinforcement learning. Nature.

[27] Chen J., Xing H., Xiao Z., Xu L., Tao T. (2021). A DRL Agent for Jointly Optimizing Computation Offloading and Resource Allocation in MEC. IEEE Internet Things J..

[28] Meng F., Chen P., Wu L., Cheng J. (2020). Power Allocation in Multi-User Cellular Networks: Deep Reinforcement Learning Approaches. IEEE Trans. Wirel. Commun..

[29] Cheng M., Li J., Nazarian S. DRL-cloud: Deep reinforcement learning-based resource provisioning and task scheduling for cloud service providers. Proceedings of the 2018 23rd Asia and South Pacific Design Automation Conference (ASP-DAC).

[30] Huang C., Mo R., Yuen C. (2020). Reconfigurable Intelligent Surface Assisted Multiuser MISO Systems Exploiting Deep Reinforcement Learning. IEEE J. Sel. Areas Commun..

[31] Pereira-Ruisánchez D., Fresnedo Ó., Pérez-Adán D., Castedo L. Joint Optimization of IRS-assisted MU-MIMO Communication Systems through a DRL-based Twin Delayed DDPG Approach. Proceedings of the 2022 IEEE International Symposium on Broadband Multimedia Systems and Broadcasting (BMSB).

[32] You C., Zhang R. (2021). Wireless Communication Aided by Intelligent Reflecting Surface: Active or Passive?. IEEE Wirel. Commun. Lett..

[33] Xu S., Du Y., Zhang J., Liu J., Wang J., Zhang J. (2023). Intelligent Reflecting Surface Enabled Integrated Sensing, Communication and Computation. IEEE Trans. Wirel. Commun..

[34] Dinh T.Q., Tang J., La Q.D., Quek T.Q.S. (2017). Offloading in Mobile Edge Computing: Task Allocation and Computational Frequency Scaling. IEEE Trans. Commun..

[35] Wang C., Liang C., Yu F.R., Chen Q., Tang L. (2017). Computation Offloading and Resource Allocation in Wireless Cellular Networks With Mobile Edge Computing. IEEE Trans. Wirel. Commun..

[36] Mao Y., Zhang J., Song S.H., Letaief K.B. (2017). Stochastic Joint Radio and Computational Resource Management for Multi-User Mobile-Edge Computing Systems. IEEE Trans. Wirel. Commun..

[37] Zhou F., Wu Y., Hu R.Q., Qian Y. (2018). Computation Rate Maximization in UAV-Enabled Wireless-Powered Mobile-Edge Computing Systems. IEEE J. Sel. Areas Commun..

[38] Feriani A., Hossain E. (2021). Single and multi-agent deep reinforcement learning for AI-enabled wireless networks: A tutorial. IEEE Commun. Surv. Tutor..

[39] Hou Y., Liu L., Wei Q., Xu X., Chen C. A novel DDPG method with prioritized experience replay. Proceedings of the 2017 IEEE International Conference on Systems, Man, and Cybernetics (SMC).

[40] Schaul T., Quan J., Antonoglou I., Silver D. (2015). Prioritized experience replay. arXiv.

[41] Fujimoto S., Hoof H., Meger D. Addressing function approximation error in actor-critic methods. Proceedings of the International Conference on Machine Learning.

[42] Zhang H., Di B., Song L., Han Z. (2020). Reconfigurable Intelligent Surfaces Assisted Communications With Limited Phase Shifts: How Many Phase Shifts Are Enough?. IEEE Trans. Veh. Technol..

[43] (2022). Study on Channel Model for Frequencies from 0.5 to 100 GHz (Release 17). Document 3GPP TR 38.901. v17.0.0. https://www.3gpp.org/DynaReport/38901.htm.

[44] Basar E., Yildirim I. (2021). Reconfigurable Intelligent Surfaces for Future Wireless Networks: A Channel Modeling Perspective. IEEE Wirel. Commun..

[45] Wang Z., Wei Y., Yu F.R., Han Z. (2022). Utility Optimization for Resource Allocation in Multi-Access Edge Network Slicing: A Twin-Actor Deep Deterministic Policy Gradient Approach. IEEE Trans. Wirel. Commun..

